# Ambulatory Status at Discharge Predicts Six-Month Mortality in Patients with COVID-19: A Retrospective Cohort Study

**DOI:** 10.3390/jcm13041129

**Published:** 2024-02-17

**Authors:** Yoonju Na, Chi Ryang Chung, Gee Young Suh, Oksoon Jeong, Ryoung-Eun Ko, Jong Geol Do

**Affiliations:** 1 Department of Physical and Rehabilitation Medicine, Samsung Medical Center, Sungkyunkwan University School of Medicine, Seoul 06351, Republic of Korea; nayoonju0201@gmail.com; 2 Department of Critical Care Medicine, Samsung Medical Center, Sungkyunkwan University School of Medicine, Seoul 06351, Republic of Korea; icu.chung@samsung.com (C.R.C.); suhgy@skku.edu (G.Y.S.); 3 Department of Data Service Team, Samsung Medical Center, Seoul 06351, Republic of Korea; os.jeong@samsung.com

**Keywords:** COVID-19, mobility, ambulation, muscle weakness, 6-months mortality

## Abstract

This retrospective cohort study aimed to evaluate the association between ambulatory status at discharge and six-month post-discharge mortality among adults with coronavirus disease (COVID-19). We analyzed data from 398 patients aged over 18 admitted to a tertiary hospital in South Korea between December 2019 and June 2022. Patients were classified into two groups based on their ambulatory status at discharge: ambulatory (able to walk independently, n = 286) and non-ambulatory (unable to walk independently, requiring wheelchair or bed-bound, n = 112). Our analysis revealed that six-month survival rates were significantly higher in the ambulatory group (94.2%) compared to the non-ambulatory group (84.4%). Multivariate analysis identified ambulatory status at discharge (*p* = 0.047) and pre-existing malignancy (*p* = 0.007) as significant prognostic factors for post-discharge survival. This study highlights that the ability to walk independently at discharge is a crucial predictor of six-month survival in COVID-19 patients. These findings emphasize the need for interventions to improve the physical performance of non-ambulatory patients, potentially enhancing their survival prospects. This underscores the importance of targeted rehabilitation and physical therapy for the comprehensive care of COVID-19 survivors.

## 1. Introduction

The outbreak of the severe acute respiratory syndrome coronavirus 2 (SARS-CoV-2), which causes coronavirus disease (COVID-19), has emerged as a global health crisis. With a staggering global death toll of 6,988,679 as of 17 December 2023, the impact of the pandemic has varied across regions. As of the latest available data, the Republic of Korea (South Korea) has reported a cumulative total of 35.9 thousand deaths due to COVID-19 [1]. The clinical spectrum of COVID-19 ranges widely, with outcomes varying from mild asymptomatic infections to critical conditions necessitating intensive care and support for multi-organ failure. Beyond the acute phase, ‘long COVID’—persistent symptoms following a severe infection—continues to impact the survivors and the healthcare system [2,3]. Recent studies indicate that COVID-19 survivors often experience various long-term physical impairments [4,5]. According to Nature’s Scientific Reports, most COVID-19 survivors reported at least one moderate-to-severe impairment, such as fatigue, muscle weakness, and difficulties in physical activity, which can significantly affect their quality of life [6]. However, the literature lacks clarity regarding the prognostic factors influencing post-hospitalization survival, particularly the physical capabilities of discharged patients. Although certain demographics and comorbidities, such as age, sex, and underlying health conditions, have been linked to COVID-19 mortality [7], the predictive value of patients’ physical status at discharge has not been adequately explored. Moreover, current prognostic assessments are complex and impractical for routine clinical use [8,9].

A study on long COVID in Korea revealed that discharged patients struggled to resume normal life because of persistent symptoms, with older individuals facing greater challenges [10]. As South Korea has navigated various pandemic phases, this study aimed to explore the relationship between ambulatory status at discharge and six-month post-discharge mortality in COVID-19 patients.

We hypothesized that the recovery of physical function after COVID-19 is an important factor in patient prognosis and that ambulatory status at discharge is closely related to the survival rate after discharge in patients with COVID-19. To better understand the clinical characteristics of patients with COVID-19, clinical factors affecting non-ambulatory status and six-month mortality were also evaluated.

## 2. Materials and Methods

### 2.1. Study Population

This retrospective cohort study included adult COVID-19 patients admitted to a single tertiary university hospital in Korea between December 2019 and June 2022. The patients were ≥18 years, had laboratory-confirmed SARS-CoV-2 infection, and had been hospitalized for at least 7 days. Patients were excluded if they died during the hospital stay or if they had a neurological disorder that could have influenced their ambulatory status. In addition, patients who were admitted for reasons unrelated to COVID-19 but later tested positive for COVID-19 prompted by the emergence of symptoms were excluded from the analysis. Our study excluded patients diagnosed with G 00-99 under ICD-10, encompassing conditions such as stroke, cerebral/cerebellar infarction, intracranial hemorrhage, spinal cord infarction, and myelopathy. This exclusion was implemented through a patient exclusion method from the Clinical Data Warehouse (CDW).

### 2.2. Data Collection

Data were extracted using our institution’s data repository (Darwin-C, Samsung Medical Center, Seoul, Republic of Korea), which automatically retrieved data from electronic medical records. Survival data post-discharge until 30 June 2022 were prospectively retrieved from the National Health Insurance Service registries. Previous medical histories were extracted from the CDW, and diagnoses were determined based on information in the patient’s admission notes, subjective, objective, assessment, and plan (SOAP) chart, and discharge notes. The attending nurse routinely recorded the ambulatory status at discharge and mobility status of the patients. Patients who could walk independently without assistance were classified as ambulatory, whereas those who could not walk independently, used a wheelchair, or were bed-bound at hospital discharge were classified as non-ambulatory. The following variables were recorded within the first 24 h of hospital and ICU admission: age, sex, weight, body mass index (BMI), pre-existing comorbidities, and vital signs, including blood pressure, O_2_ saturation, heart rate, and respiratory rate. Data on the length of stay (LOS), preexisting comorbidities, newly diagnosed comorbidities, and initial laboratory results within 48 h of admission (creatinine, D-dimer, procalcitonin, lactic acid, C-reactive protein [CRP], and alkaline phosphatase [ALP] levels) were extracted from electronic medical records. Comorbidities were traced using the International Classification of Diseases (ICD)—10th revision codes. The following ICU-related variables were also extracted from the records: length of ICU admission; use of extracorporeal membrane oxygenation; use of corticosteroids, neuromuscular blocking agents, and amiodarone; initial Sequential Organ Failure Assessment (SOFA) score; maximum SOFA score; and initial Richmond Agitation–Sedation Scale score.

### 2.3. Statistical Analysis

The categorical variables were analyzed using Pearson’s chi-squared test. Continuous variables were analyzed using independent sample *t*-tests and Mann–Whitney–Wilcoxon U-tests to assess differences in the distribution of variables between the groups. After checking the histogram of each variable, a two-sample *t*-test or Wilcoxon rank-sum test was performed to determine the differences in mean or median values between the groups. We calculated the six-month survival probabilities using Kaplan–Meier survival estimates and used the log-rank test to compare the survival of the two groups (ambulatory and non-ambulatory patients). Survival status was updated using regional health authority records and determined for all patients as of 30 June 2022.

Associations between the risk factors were analyzed using univariate and multivariate logistic regression models. Variables with *p* values < 0.05 in the univariate analyses were subjected to multivariate analysis. Multivariate Cox regression analysis was performed to determine whether ambulatory status at discharge was significantly associated with survival after discharge. Covariates with *p* < 0.05 in univariate analysis were entered into the multivariable model. Our final model was fitted based on multiple imputed datasets using Rubin’s rules to combine effect estimates and standard errors to allow for the uncertainty caused by missing values. *p* values < 0.05 were considered statistically significant. Data analyses were performed using the R software (version 3.6.3; Foundation for Statistical Computing, Vienna, Austria).

## 3. Results

**Figure 1:**
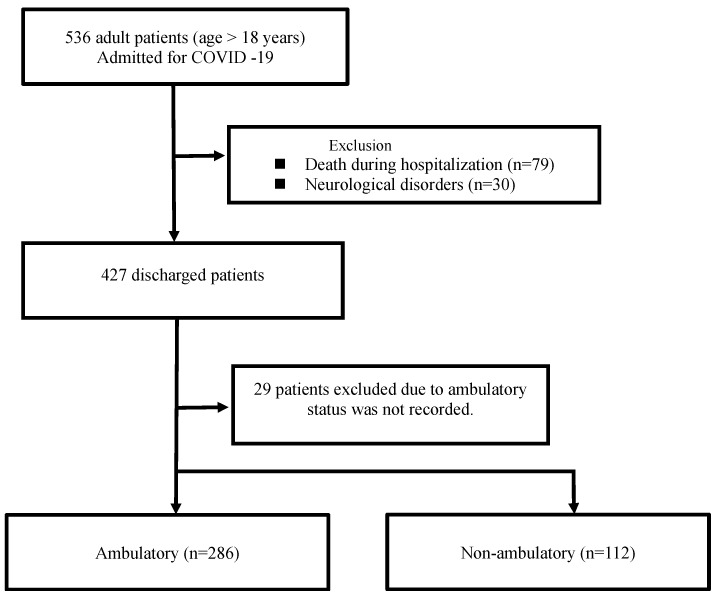
Flow diagram of patient selection.

A total of 536 patients aged 18 years and older, who had been hospitalized for more than 7 days with confirmed COVID-19, were identified. Among them, 79 died prior to discharge, and 30 were excluded due to the presence of a pre-existing neurological disorder that could influence their ambulatory status. Of the 427 discharged patients, 29 were excluded because their ambulatory status was not recorded by the attending nurse (Figure 1).

### 3.1. Patient Characteristics

A total of 398 patients were included in the analysis, with 228 (57.3%) men and 170 (42.7%) women, with a mean age of 62.74 ± 15.40 years. At hospital discharge, 286 patients (71.9%) were ambulatory, and 112 patients (28.1%) were non-ambulatory (Table 1).

Compared with patients in the ambulatory group, patients in the non-ambulatory group were significantly older (mean age 59.8 ± 15.4 years vs. mean age 69.2 ± 14.0 years; *p* < 0.0001) and had a longer LOS (median: 14 [IQR: 9–22.75] days vs. median: 19 [IQR: 11–37] days; *p* = 0.001). Regarding medication use, patients in the non-ambulatory group were more likely to be treated with corticosteroids (*p* = 0.004) and neuromuscular blocking agents (*p* = 0.047) than those in the ambulatory group. A total of 117 out of the entire cohort were treated in the Intensive Care Unit (ICU), with 78 patients (27.3%) in the ambulatory group and 39 patients (34.8%) in the non-ambulatory group (*p* = 0.173).

### 3.2. Clinical Factors Associated with Six-Month Survival

**Figure 2:**
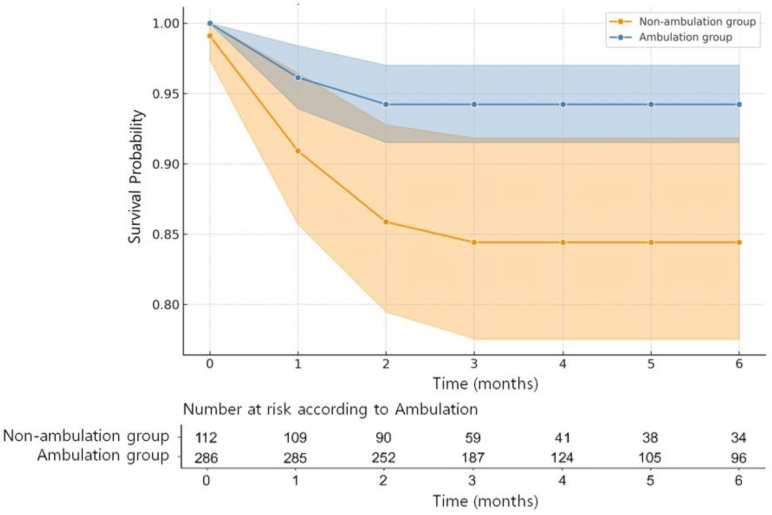
Kaplan–Meier estimate of the six-month survival probabilities with 95% confidence intervals for ambulatory group versus non-ambulatory group. The ambulatory group had better survival over time (log-rank test *p* = 0.003). The shaded regions around each survival curve represent the 95% confidence intervals.

The Kaplan–Meier plot showed a significant difference in hospital survival between the two groups (*p* = 0.003) (Figure 2). The six-month overall survival rates were 91.5% in the study population and 94.2% and 84.4% in the ambulatory and non-ambulatory groups, respectively. Notably, 33 patients died, with 16 (5.6%) in the ambulatory group and 17 (15.2%) in the non-ambulatory group (*p* = 0.004).

In the multivariate analysis of six-month mortality, ambulatory status at hospital discharge (adjusted hazard ratio [HR]: 2.65, 95% confidence interval [CI]: 1.10–6.39, *p* = 0.047) and presence of malignancy (adjusted HR: 5.25, 95% CI: 1.88–14.69, *p* = 0.007) were independent predictors of six-month mortality (Table 2). Notably, previous disease severity and laboratory findings at admission were not associated with an increased six-month mortality.

### 3.3. Clinical Factors Associated with Ambulatory Status at Discharge

Factors associated with an increased risk of non-ambulatory status at hospital discharge were identified using logistic regression analysis. In the multivariable analysis, age >65 years (adjusted odds ratio [OR]: 3.28, 95% CI: 1.94–5.56, *p* < 0.0001), longer LOS (adjusted OR: 1.67, 95% CI: 1.22–2.28, *p* = 0.002), and the use of corticosteroid during hospitalization (adjusted OR: 1.74, 95% CI: 1.01–2.99, *p* = 0.046) were significantly associated with ambulatory status at discharge (Table 3).

## 4. Discussion

We found that ambulatory status at hospital discharge was a significant predictor of six-month mortality. This is consistent with the findings of the study conducted by Pamela et al. [11] in Spain. They measured the Barthel Index in older COVID-19 patients and found it to be associated with six-month mortality. This Spanish study highlighted the significant relationship between functional status and outcomes in older COVID-19 patients. According to them, the incidence of posthospitalization functional impairment 6 months after discharge was 41.7% higher than that described at 3 months (27.2%), which could be related to dyspnea as a persistent symptom limiting ambulation and the performance of basic activities of daily living. We selected a simpler measure of functional status at discharge using the patients’ ambulation ability as an indicator, which was also found to be associated with six-month mortality. This holds clinical significance as it offers a more straightforward approach. Our findings are consistent with data from a UK study [12] highlighting the prevalence of mobility problems in COVID-19 patients. This study emphasized the long-term health impacts of COVID-19, particularly on mobility, and further validated our focus on ambulatory status as a crucial indicator of patient outcomes. This observation aligns with recent findings from a Canadian study that reported significant mobility limitations and activities of daily living (ADL) impairment in older COVID-19 patients, along with a higher incidence of severe muscle weakness compared with non-infected older individuals [13].

A recent systematic review reported that male sex, older age, leukocytosis, cardiac injury, and high-dose corticosteroid treatment were associated with a high risk of COVID-19-related mortality [14,15,16,17,18]. Among patients with COVID-19, the reported mortality rate varies widely from 5% to 78% [15,19,20,21]. This study distinguishes itself from previous research by identifying factors associated with the six-month mortality rate. Specifically, the patient’s ambulatory status at discharge and the presence of an underlying malignancy were significant factors that influenced the mortality rate. This wide disparity can be explained by geographical variations by country, changes in vaccination coverage, and predominant SARS-CoV-2 variants over time. The mortality rate in our study differs from that in other studies because of the differences in the study setting. Herein, we report mortality rates only for patients who were discharged alive and died within six months of discharge.

Older age (>65 years), prolonged hospital LOS, and corticosteroid use during admission were significantly associated with non-ambulatory status at discharge. Drawing parallels with the 2003 outbreak of severe acute respiratory syndrome (SARS), the proinflammatory effects of viral infection and deconditioning during the convalescent period have been suggested as key factors contributing to deficits in both muscle strength and endurance [22,23]. In patients with COVID-19, physical impairment and muscle weakness can be attributed to multiple factors, potentially arising from infection, induced systemic inflammation, changes in myokine production, and decreased physical activity [24]. Sarcopenia and skeletal muscle changes following COVID-19 can increase the vulnerability to post-COVID-19 functional and physical decline. Our study identified three factors that can influence these changes: older age (>65 years), prolonged LOS, and the use of corticosteroids. Muscle weakness is common in patients, particularly those with prolonged hospitalization. Furthermore, muscle weakness and decreased physical function may be more prevalent in critically ill patients. In our study, the non-ambulatory group had an average LOS of 19 days, which was 5 days longer than that of the ambulatory group. In this study, the non-ambulatory group had a higher proportion of ICU admissions, which are associated with the development of neuropathy and/or myopathy, known as ICU-acquired weakness (ICU-AW). This condition can be attributed to respiratory issues in COVID-19 patients, resulting in a decrease in muscle cross-sectional area and muscle fiber size [25]. Limb muscle weakness in COVID-19 patients is significantly associated with factors such as prolonged prone positioning, catecholamine use, and the duration of mechanical ventilation [26]. Low-dose corticosteroid therapy (e.g., dexamethasone) is frequently used to treat patients with severe COVID-19, whereas high-dose corticosteroid therapy is generally not recommended because of its harmful effects [27]. Corticosteroid therapy can alter specific gene expression, inhibiting protein synthesis and muscle wasting [28]. In patients with COVID-19, the use of high-dose corticosteroids contributes more to poor clinical outcomes than the initiation time or duration of systemic corticosteroid use [16]. Although corticosteroids have been found to be beneficial in treating patients with COVID-19, high-dose use may have negative consequences, including muscle weakness. It is necessary to better understand the natural course of muscle weakness after COVID-19 and carefully consider the appropriate use of high-dose corticosteroids.

A systematic review reported that the recovery of physical function following COVID-19 is incomplete and that impaired physical function can persist for up to 2 years [29]. In a one-year follow-up study in China [30], it was reported that the majority of individuals under the age of 65 returned to life without disability 1 year later, with good physical and functional recovery. However, it has become evident that respiratory infections, such as COVID-19, can result in physical limitations, especially in vulnerable groups such as the older population and patients with cancer, and this issue is linked to increased mortality rates. In our study, most patients were individuals aged 65 and older. Older non-ambulatory patients discharged from hospitals require post-discharge care and proper rehabilitation to recover their physical function.

Rehabilitation therapy was found to be feasible and could improve physical and cognitive function in patients recovering from COVID-19 [31]. One study reported that of 16 patients who had muscle weakness, seven (44%) could not walk 100 m, even after 30 days of weaning. Among the nine patients who underwent early rehabilitation after ICU discharge, only one patient was unable to walk 30 days later, suggesting the potential for recovering walking ability in patients with COVID-19 admitted to the ICU [26]. Several rehabilitation protocols for long-term COVID-19 symptoms have been reported [32,33,34,35]. Future studies should evaluate a larger number of patients undergoing rehabilitation and examine the effects of rehabilitation on mortality. Our study conducted a comprehensive analysis of approximately 400 patients, which is significant because of the large sample size. The analysis covered various clinical factors, including vital signs upon admission and included patients with underlying disease conditions. Furthermore, this study confirmed the association between ambulatory discharge status and survival rate. In addition, patients aged >65 years represent a vulnerable population with a decline in physical function following infection. The significance of this finding lies in the identification of a population that should be considered for rehabilitation therapy and other interventions during hospitalization. This can serve as evidence supporting the need for interventions to improve patient mobility during hospitalization. Therefore, clinicians should consider the potential association between functional impairment and decreased physical function related to infectious diseases and survival rates in future studies.

This study has several limitations. First, it was conducted at a single tertiary hospital in South Korea. Second, this was a retrospective study based on the data available from the CDW. Owing to its design, our study may have inherent biases related to residual confounding factors and a lack of randomly distributed exposure. Third, ambulatory status was only assessed at discharge, and we could not compare discharge and prehospital ambulatory status or adjust for prehospital ambulatory status, which may have affected the ambulatory status at discharge. Fourth, because we used ICD codes to extract data on pre-existing comorbidities, the malignancy type could not be determined. Furthermore, we could not perform a survival analysis for SARS-CoV-2 variants because our hospital did not test for variants.

## 5. Conclusions

Ambulatory status at discharge could predict the six-month mortality of patients with COVID-19. Efforts during hospitalization are important for maintaining and improving patients’ physical function. Furthermore, prolonged hospitalization, old age, and corticosteroid use during hospitalization were associated with the deterioration of ambulatory function at discharge, suggesting the need for physical rehabilitation to improve physical function among these patients. This study suggests potential avenues for future research, such as investigating the impact of mobilization and rehabilitation on survival rates.

## Figures and Tables

**Table 1:** Baseline characteristics of study population.

	Ambulatory (N = 286)	Non-Ambulatory (N = 112)	Total (N = 398)	*p*-Value
Patients’ characteristics				
Age, mean (±SD), years	59.82 (±15.13)	69.22 (±14.02)	62.47 (±15.40)	<0.0001 ^a^
Age >65 years, n (%)	130 (45.5)	81 (72.3)	211 (53.0)	<0.0001 ^a^
Male, n (%)	162 (56.6)	66 (58.9)	228 (57.3)	0.7628
Length of stay, median (IQR), day	14 (9–22.75)	19 (11–37)	15 (10–27.75)	0.0008 ^a^
Body mass index, mean (±SD), kg/m^2^	23.82 (4.42)	22.70 (4.51)	23.51 (4.47)	0.0364 ^b^
Body mass index—group				
Underweight, <18.5, n (%)	24 (8.4)	12 (12.2)	36 (10.1)	0.0706
Normal, <23, n (%)	90 (31.5)	45 (45.9)	135 (38.0)	
Overweight, <25, n (%)	55 (19.2)	11 (11.2)	66 (18.6)	
Obese, ≥25, n (%)	88 (30.8)	30 (30.6)	118 (33.2)	
Previous medical history				
Hypertension, n (%)	55 (19.2)	30 (26.8)	85 (21.4)	0.1291
Diabetes mellitus, n (%)	48 (16.8)	29 (25.9)	77 (19.3)	0.05388
Osteoporosis, n (%)	10 (3.5)	4 (3.6)	14 (3.5)	1.0000
Coronary artery disease, n (%)	1 (0.3)	0 (0)	1 (0.3)	1.0000
Immunosuppression, n (%)	8 (2.8)	3 (2.7)	11(2.8)	1.0000
Malignancy, n (%)	110 (38.5)	40 (35.7)	150 (37.7)	0.6939
Chronic kidney disease, n (%)	35 (12.2)	11 (9.8)	46 (11.6)	0.6145
Heart failure, n (%)	15 (5.2)	11 (9.8)	26 (6.5)	0.1510
Chronic obstructive pulmonary disease, n (%)	9 (3.1)	5 (4.5)	14 (3.5)	0.7346
Initial laboratory findings, vital signs at admission				
Creatinine, median (IQR), mg/dL	0.77 (0.59–1.08)	0.83 (0.6–1.3)	0.79 (0.59–1.12)	0.4370
D-dimer median (IQR), µg/mL	1.42 (0.68–4.14)	1.83 (1.03–3.68)	1.58 (0.78–3.97)	0.2849
Procalcitonin, median (IQR), mol/L	0.16 (0.06–0.62)	0.20 (0.09–0.84)	1.85 (0.07–0.64)	0.0563
Lactic acid, median (IQR), mmol/L	1.41 (1.01–1.98)	1.50 (1.07–2.10)	1.44 (1.04–2.04)	0.3492
C-reactive protein, mean (±SD), mg/dL	7.57 (8.05)	9.36 (8.89)	8.07 (8.32)	0.0722
Alkaline phosphatase (IQR), U/L	75.5 (58.25–101)	81 (67–104.5)	78 (61–103)	0.0407 ^b^
Systolic blood pressure, mean (±SD), mmHg	126.45 (24.37)	127.75 (23.50)	126.82 (24.10)	0.6243
Diastolic blood pressure, mean (±SD), mmHg	76.90 (14.53)	73.55 (14.86)	75.95 (14.68)	0.0436 ^b^
Oxygen saturation, mean (±SD), %	94.80 (7.00)	93.62 (7.62)	94.47 (7.19)	0.1599
Heart rate/min, mean (±SD)	89.80 (19.61)	92.44 (22.24)	90.56 (20.40)	0.3365
Respiratory rate/min, mean (±SD)	20.43 (4.72)	21.67 (5.53)	20.79 (4.98)	0.0387 ^b^

Abbreviations: SD, standard deviation; n, number; IQR, interquartile range. ^a^ *p* < 0.01, ^b^ *p* < 0.05.

**Table 2:** Cox proportional hazards regression analyses for overall six-month mortality.

	Univariate		Multivariate	
	Crude HR (95% CI)	*p*-Value	Adjusted HR (95% CI)	*p*-Value
Patients’ characteristics				
Age	1.01 (0.99–1.04)	0.293		
Gender, female	0.50 (0.23–1.08)	0.076		
LOS	1.36 (0.92–2.02)	0.127		
Ambulation at discharge, non-ambulation status	2.74 (1.37–5.47)	0.004	2.65 (1.10–6.39)	0.047
BMI	0.91 (0.84–1.00)	0.054		
Previous medical history				
Hypertension	0.70 (0.27–1.81)	0.459		
Diabetes mellitus	0.59 (0.21–1.68)	0.320		
Osteoporosis	0.0000	0.997		
Coronary artery disease	0.0000	0.997		
Immunosuppression	2.44 (0.58–10.19)	0.223		
Malignancy	7.80 (3.21–18.96)	<0.0001	5.25 (1.88–14.69)	0.007
Chronic kidney disease	0.86 (0.26–2.81)	0.797		
Heart failure	0.95 (0.23–3.96)	0.939		
Chronic obstructive pulmonary disease	1.03 (0.14–7.54)	0.977		
Initial laboratory findings and vital signs at admission				
Creatinine	0.87 (0.51–1.50)	0.615		
D-dimer	1.33 (0.91–1.94)	0.137		
Procalcitonin	1.28 (1.04–1.56)	0.017	1.09 (0.84–1.42)	0.535
Lactic acid	1.85 (1.01–3.40)	0.047	1.76 (0.84–3.56)	0.136
C-reactive protein	1.33 (1.01–1.75)	0.040	1.09 (0.76–1.55)	0.656
Alkaline phosphatase	2.66 (1.82–3.91)	<0.0001	1.52 (0.88–2.63)	0.156
SBP	0.98 (0.97–1.00)	0.033		
DBP	0.99 (0.96–1.01)	0.239		
Saturation	10.44 (0.04–3026.58)	0.417		
Heart rate	1.02 (1.00–1.03)	0.065		
Respiratory rate	0.96 (0.89–1.04)	0.304		
Medication administered during hospitalization				
Corticosteroid	1.44 (0.69–2.99)	0.328		
Neuromuscular blocking agent	0.82 (0.20–3.43)	0.785		
Amiodarone	2.54 (0.61–10.64)	0.202		
ICU characteristics				
Admission at ICU	0.69 (0.30–1.59)	0.379		
Number of ICU admissions during stay	1.57 (0.27–9.16)	0.619		
LOS in ICU	1.14 (0.69–1.88)	0.604		
Maximum SOFA	1.28 (0.17–3.50)	0.634		
RASS score	1.10 (0.78–1.55)	0.574		
ECMO	0.0000	0.999		
Rehabilitation during ICU	0.64 (0.15–2.67)	0.538		
Number of rehabilitations	0.57 (0.28–1.19)	0.137		

Abbreviations: HR, hazard ratio; CI, confidence interval; BMI, body mass index; ICU, intensive care unit; SBP, systolic blood pressure; DBP, diastolic blood pressure; LOS, length of stay; SOFA, Sequential Organ Failure Assessment; RASS, Richmond agitation–sedation scale; ECMO, extracorporeal membrane oxygenation.

**Table 3:** Clinical factors associated with non-ambulatory status in patients at discharge.

	Univariate		Multivariate	
	Crude OR (95% CI)	*p*-Value	Adjusted OR (95% CI)	*p*-Value
Patients’ characteristics				
Age	1.05 (1.03–1.07)	<0.0001		
Age > 65 years	3.14 (1.97–5.10)	<0.0001	3.28 (1.94–5.56)	<0.0001
Gender, female	0.91 (0.58–1.42)	0.679		
LOS	1.64 (1.25–2.16)	0.000	1.67 (1.22–2.28)	0.002
BMI	0.94 (0.89–0.99)	0.035		
Previous medical history				
Hypertension	1.54 (0.92–2.55)	0.100		
Diabetes mellitus	1.73 (1.02–2.92)	0.040	1.61 (0.90–2.87)	0.111
Osteoporosis	1.02 (0.28–3.13)	0.971		
Coronary artery disease	0.78 (0.37–1.55)	0.981		
Immunosuppression	0.96 (0.21–3.38)	0.948		
Malignancy	0.89 (0.56–1.39)	0.611		
Chronic kidney disease	0.78 (0.37–1.55)	0.499		
Heart failure	1.97 (0.86–4.40)	0.102		
Chronic obstructive pulmonary disease (COPD)	1.44 (0.43–4.26)	0.523		
Initial laboratory findings and vital signs at admission				
Creatinine (log)	0.97 (0.69–1.32)	0.835		
D-dimer (log)	1.06 (0.85–1.33)	0.612		
Procalcitonin (log)	1.17 (1.01–1.37)	0.042	1.09 (0.93–1.28)	0.278
Lactic acid (log)	1.21 (0.79–1.85)	0.384		
C-reactive protein (CRP),(log)	1.18 (1.02–1.37)	0.029	0.98 (0.83–1.17)	0.821
Alkaline phosphatase (ALP), (log)	1.29 (0.86–1.94)	0.215		
SBP	1.00 (0.99–1.01)	0.629		
DBP	0.98 (0.97–1.00)	0.042		
Saturation (log)	0.26 (0.03–2.48)	0.229		
Heart rate	1.01 (0.99–1.02)	0.310		
Respiratory rate	1.05 (1.01–1.09)	0.028	1.04 (0.99–1.09)	0.137
Medication given during hospitalization				
Corticosteroid	2.02 (1.28–3.24)	0.003	1.74 (1.01–2.99)	0.046
Neuromuscular blocking agent	2.26 (1.06–4.76)	0.032	1.64 (0.71–3.82)	0.251
Amiodarone	2.64 (0.81–6.62)	0.099		
Intensive care unit (ICU) characteristics				
Admission to ICU	1.43 (0.89–2.27)	0.138		

BMI, Body mass index; CI, confidence interval; ICU, intensive care unit; SBP, systolic blood pressure; DBP, diastolic blood pressure; LOS, length of stay; SOFA, Sequential Organ Failure Assessment; RASS, Richmond Agitation–Sedation Scale; ECMO, extracorporeal membrane oxygenation; OR, odds ratio.

## Data Availability

Data are available upon reasonable request.
